# Long-Term Impact of COVID-19 Pandemic in Sleep Quality and Lifestyle in Young Adults

**DOI:** 10.3390/ijerph191912333

**Published:** 2022-09-28

**Authors:** Argyrios Eleftherios Barbouzas, Foteini Malli, Zoe Daniil, Konstantinos Gourgoulianis

**Affiliations:** 1 Department of Respiratory Medicine, Faculty of Medicine, University of Thessaly, Biopolis, 41110 Larissa, Greece; 2 RespiratoryDisorders Laboratory, Faculty of Nursing, School of Health Sciences, University of Thessaly, Gaiopolis, 41110 Larissa, Greece

**Keywords:** COVID-19, quarantine, young adults, lifestyle, sleep, smoking, eating, physical activity

## Abstract

Due to the evolution of COVID-19,restrictive measures were implemented. The quarantine resulted in significant changes in the social, economic, and psychological status of the population; however, its long-term effects have not yet been elucidated, especially in young adults. In the present study, people aged 18–25 years were studied, in relation to their sleeping, smoking, eating, and drinking habits and their physical activity before, during, and after the implementation of quarantine. We included 540 respondents (21.2 ± 2.3 years, 62.8% female). During quarantine, from 23 March to 4 May 2020, we observed an increase in sleep hours by 1.17 ± 1.98 h (*p* < 0.001), time of sleep arrival by 11.90 ± 30.41 min (*p* < 0.001), and number of daily meals by 0.66 ± 1.4 (*p* < 0.001). The subjects who consumed alcoholic beverages never or almost never had an upward change of 27.04%, and the time of daily exercise was reduced by 10.16 ± 48.68 min (*p* < 0.001). After quarantine, cigarettes per day increased by 1.14 ± 3.62 (*p* < 0.001) and the awakenings during night time increased by 0.37 ± 1.93 (*p* < 0.001). Our results suggest that the quarantine brought about significant changes in smoking, sleeping habits, physical activity, dietary habits, and the consumption of alcoholic beverages, some of which continue after its termination.

## 1. Introduction

In December 2019 a new coronavirus strain [1] causing COVID-19 [2] led the WHO to declare a pandemic [3]. The first case of COVID-19 in Greece was recorded in February 2020 [4] and the Greek government, while initially managing to limit the evolution of the pandemic [5], finally implemented community and public health measures (quarantine) from 23 March to 4 May 2020, in order to flatten the epidemic curve and reduce the load imposed on the Greek healthcare system [6]. Although the prolonged social isolation reduced the overload of public health facilities, the imposed quarantine, in addition to the fear of death and disease, may contribute to psychological adverse events [7].

During the quarantine, people were faced with many and important changes in their lives and in their daily routines, with significant social [8], economic [9], and mental effects [10], and at the same time a reduction in the provision of health services [11]. There are now health concerns, the fear of being infected with the virus [12], anxiety about the future of work [13], loneliness [14], and social isolation [15] but also more time at home [16]. Suspension in the use of workplaces, restaurants, gyms, and other public places has led to changes in eating habits [17], physical inactivity [18], consumption of tobacco products [19], and increased alcohol consumption [20]. Changes were also observed in sleep habits [21,22], with significant effects on duration and sleep quality, resulting in promotion of sleep disturbances [23]. Despite the extensive research, the literature has only scarce data about the long-term impact of restrictive measures due to COVID-19 on lifestyle changes. Interestingly, the behavior of young adults during and after quarantine has not been extensively studied in the literature. 

The lifestyle changes (including changes in physical activity and diet) that occurred during quarantine may have a long-term impact on health; restrictive measures may carry a long-term increase in the risk of cardiovascular disease [24]. In the same context, lockdown, in tandem with the COVID-19 pandemic itself, may adversely impact the control of non-communicable diseases (i.e., risk factors, symptoms, and management) [25]. In addition, children and adolescents may experience long-term psychological effects due to the COVID-19 pandemic [26]. The long-term effects of quarantine, especially in poorly examined populations, require further investigation in order to effectively organize public health programs aimed at the restoration of health following the pandemic. 

In the present study, we aimed to assess the long-term effects of restrictive measures on an underrepresented population in the literature (i.e., young adults). To this end, young people, aged 18–25 years, were studied regarding smoking, sleeping habits and behaviors, consumption of meals, drinks, and cigarettes, and physical activity-exercise, in order to investigate the changes that may occur as a result of quarantine enforcement measures.

## 2. Materials and Methods

### 2.1. Study Design

This is a retrospective cohort observational study that used data collected using an online survey that included all regions of Greece and was conducted from 2 September to 27 November 2020. The study concerned young adults, aged 18–25 years, residents of Greece. Participants were asked to answer a questionnaire that was structured using the Google Form website The full questionnaire has been provided as supplementary material (Table S1). A total of 558 participants completed the questionnaire. Of those, 18 had invalid data and were excluded from the study.

The questionnaire was distributed through social media. This method was effective in terms of geographical distribution taking into account the restrictive measures due to the pandemic, mainly due to the systematic involvement of young people with the Internet [27,28]. 

The questionnaire included a total of 62 questions divided into 5 different sections. The first section (6 questions) included demographics, the second section (17 questions) addressed smoking habits, the third section was about sleep–wake habits, the fourth section (6 questions) assessed diet and alcohol consumption habits, and the fifth section concerned physical activity and exercise (9 questions). The questions in Section 2, Section 3, Section 4 and Section 5 concerned three phases, PRE (3 months prior to lockdown), DURING, and POST (3 months post-lockdown) the implementation of the restrictive measures. All questions were mandatory and there were section shortcuts where needed (e.g., smoking questions were not displayed to non-smokers). 

A pilot test of the first draft of the questionnaire was performed with a limited number of participants in order to assess ability to complete the questionnaire. We estimated that the time required for the completion of the questionnaire was about 10–20 min. We modified the questionnaire according to the results of the pilot testing. We did not include the responses of the draft questionnaire in the final analysis.

### 2.2. Statistical Analysis

Data are presented as mean and standard deviation (mean ± SD), frequencies (N), and percentages (%). Body Mass Index (BMI) categories was classified as follows: (1) underweight (<18.5 kgr/m^2^), (2) normal weight (18.5–24.9 kgr/m^2^), (3) overweight (25–29.9 kgr/m^2^), (4) obese (≥30 kgr/m^2^). Smokers were classified according to their smoking habit depending on the number of cigarettes per day (NOΝ SMOKERS, <5, 5–10, >10, cigarettes per day). Additionally, subjects were classified according to the hours of sleep (<7, 7–9, >9 h) and the time of sleep arrival (<10, 10–15, >15 min). All variables were tested for regularity according to the criteria of the Kolmogorov–Smirnov test. Bivariate correlations were performed with the use of the Pearson coefficient or Spearman coefficient, according to the distribution of the variables. In the cases of normality, the *t*-test was used for the statistical comparison between groups and the paired-samples *t*-test or ANOVA for variables, which were measured in the same individuals PRE, DURING, and POST the mobility restrictions. In the cases of non-normality, we used the Mann–Whitney U, Kruskal Wallis, Wilcoxon z, and the Friedman test, respectively. The correlations between nominal variables were calculated by the chi-square test (x^2^) and Cramer’s V [29]. The variables assessed POST-lockdown were tested with linear regression analysis. Variables with a significance level below <0.05 were retained for the analysis. The coefficient of determination (r^2^) was used to estimate the percentage of effect explained by the model. The level of statistical significance was set at *p* < 0.05. The statistical analysis was performed with the statistical program SPSS, version 20 (SPSS Inc., Chicago, IL, USA). 

## 3. Results

### 3.1. Demographic Characteristics

The online survey was completed from 2 September 2020 to 27 November 2020. The mean age of the subjects included was 21.2 ± 2.3, years. Of the study population, 37.2% were men and 30.7% had higher BMI than normal. The demographic characteristics of the subjects included are presented in detail in Table 1. 

**Figure 1:**
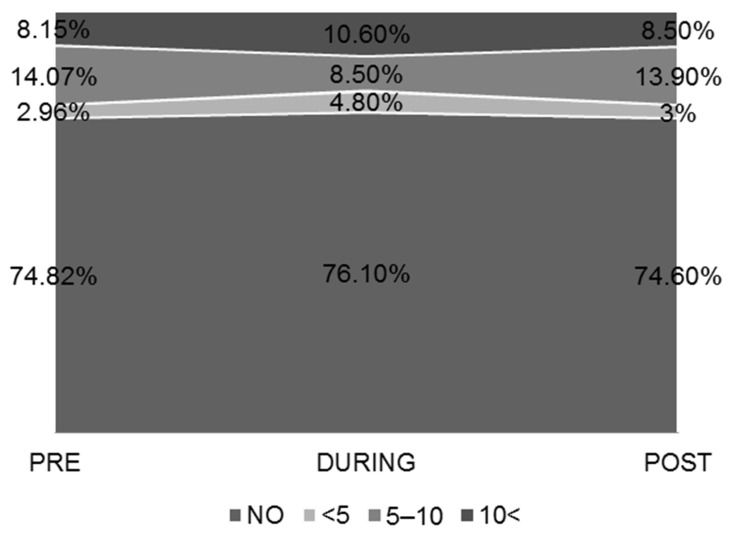
Smoking habits of the study population.

Smoking history of smokers is shown in detail in Table 2. Approximately ¼ (25.5%) of the participants were smokers. We observed a significant difference in Pack-Years between men and women (*p* = 0.001). When we compared the smoking habits before the lockdown with the smoking habits during the lockdown there was no significant change in the total of subjects. However, when comparing the PRE to the POST phase there was an increase of cigarette consumption by 1.14 ± 3.62 (*p* < 0.001) in the total of subjects. DURING quarantine, the percentage of young men and women who smoked 5–10 cigarettes per day decreased, as opposed to the percentage of those who smoked <5 and >10 cigarettes per day which increased. The percentage of non-smokers remained relatively stable (Figure 1). Consumption of vaping did not show a statistically significant difference between the quarantine phases. 

When we stratified participants by gender, we observed that the number of cigarettes per day showed a significant difference between the two sexes in all three phases of quarantine (PRE, *p* < 0.001, DURING, *p* = 0.009, POST, *p* = 0.005) (Table 3). Additionally, obese subjects showed a significant increase in the number of cigarettes per day both in the PRE vs. DURING (9.67 ± 6.4 vs. 12.83 ± 7.98, respectively, *p* = 0.042, Wilcoxon z) and the PRE vs. POST phases (12.33 ± 7.05, respectively, *p* = 0.026, Wilcoxon z). Moreover, there was a significant increase in the number of cigarettes per day of 1.54 ± 3.84 cigarettes/day (*p* = 0.001, Wilcoxon z) and of 1.5 ± 3.22 cigarettes/day (*p* < 0.001, Wilcoxon z) in women and in normal weight subjects, respectively. 

### 3.2. Sleep–Wake Habits

**Figure 2:**
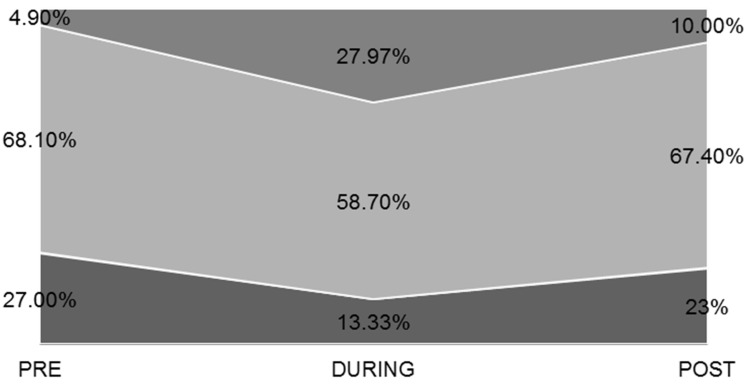
Hours of sleep.

Sleeping hours in young adults PRE versus DURING lockdown increased by 1.17 ± 1.98 h (7.29 ± 3.56 vs. 8.47 ± 1.89, respectively, *p* < 0.001, Wilcoxon z) in the whole group. POST-lockdown sleeping hours increased by 0.27 ± 1.52 h (7.29 ± 3.56 vs. 7.56 ± 1.49, respectively, *p* < 0.001, Wilcoxon z). DURING quarantine, the percentage of those who slept >9 h increased by 23.07% while after lockdown it increased by 5.1% (Figure 2).

We stratified the sample according to gender and observed that in men, sleeping hours increased by 1.04 ± 1.82 h (7.29 ± 1.30 vs. 8.34 ± 1.86, respectively, *p* < 0.001, Wilcoxon z) and in women by 1.25 ± 2.07 h (7.29 ± 1.35 vs. 8.54 ± 1.90, respectively, *p* < 0.001, Wilcoxon z). when taking BMI into account, we demonstrated that POST-lockdown sleeping hours increased by 0.84 ± 2.02 h (7.08 ± 1.47 vs. 7.92 ± 1.77, respectively, *p* = 0.013, Wilcoxon z) in underweight subjects, and increased by 0.26 ± 1.39 h (7.32 ± 1.27 vs. 8.52 ± 1.89, respectively, *p* = 0.001, Wilcoxon z) in normal weight participants. 

**Figure 3:**
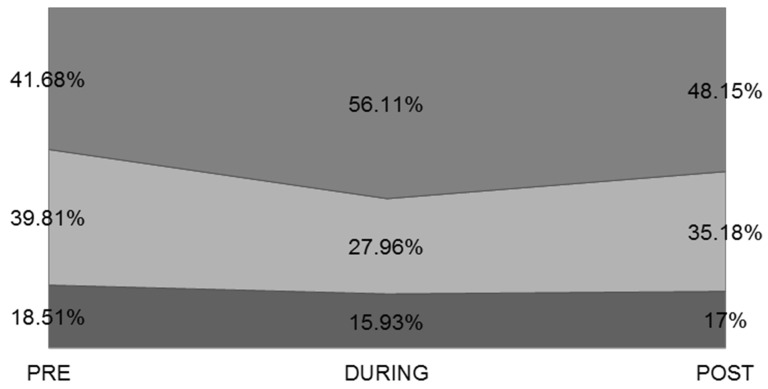
Sleep arrival during quarantine (minutes).

A significant change was found in the total group, in the comparison of PRE vs. DURING phase (20.42 ± 18.26 vs. 32.32 ± 35.22, minutes, respectively, *p* < 0.001, Wilcoxon z) and in the PRE vs. POST phase (20.42 ± 18.26 vs. 24.48 ± 23.99, minutes, respectively, *p* < 0.001, Wilcoxon z). The percentage of the study population with sleep arrival >15 min, increased by 14.43% DURING quarantine and by 6.47% POST-quarantine (Figure 3). When accounting for gender, we observed that the time of sleep arrival between men and women was significantly different DURING the quarantine (26.48 ± 26.48 vs. 35.79 ± 38.95, minutes, respectively, *p* = 0.008, Wilcoxon z). 

A significant difference in sleep awakenings was found in the total group, in the PRE vs. DURING phase (0.88 ± 1.95 vs. 1.21 ± 1.54, respectively, *p* < 0.001, Wilcoxon z) and in the PRE vs. POST phase (0.88 ± 1.95 vs. 1.26 ± 1.42, respectively, *p* < 0.001, Wilcoxon z). Sleep awakenings between men and women demonstrated a significant difference DURING the quarantine (0.93 ± 1.26 vs. 1.38 ± 1.66, respectively, *p* = 0.002, Mann–Whitney U) and POST-quarantine (1.02 ± 1.24 vs. 1.40 ± 1.51, respectively, *p* = 0.003, Mann–Whitney U). Regarding restless sleep, difficulty in getting up, morning headaches, and morning irritability, in all phases of the pandemic, there was a significant difference between men’s and women’s percentages (Table 4). 

**Figure 4:**
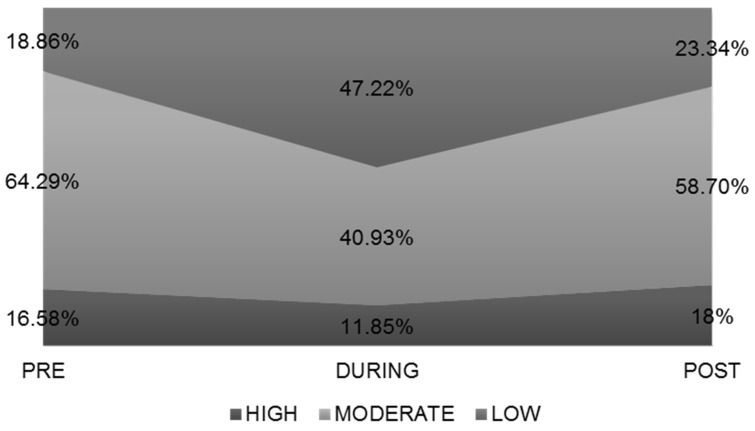
Level of morning alertness (categories).

In the period DURING quarantine, the percentage of young people with low morning alertness increased by 28.36% and by 4.48% POST-quarantine (Figure 4). There was a significant difference between men’s and women’s percentages (PRE (φ_c_ = 0.707, *p* < 0.001), DURING (φ_c_ = 0.712, *p* < 0.001), POST (φ_c_ = 0.708, *p* < 0.001), Cramer’s V) (Table 5). 

### 3.3. Dietary Habits and Consumption of Alcoholic Beverages

**Figure 5:**
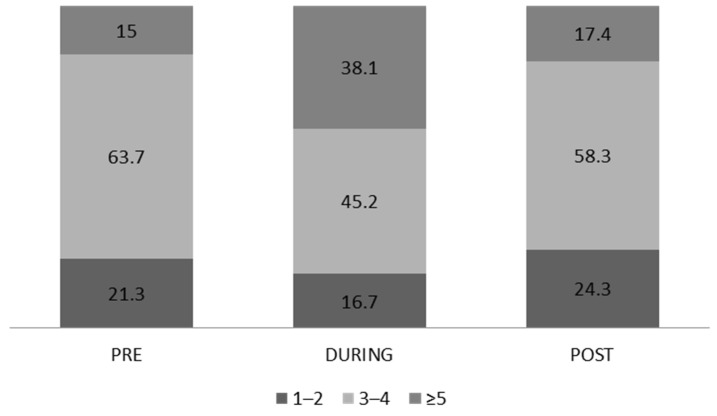
Number of meals per day in the study population.

We observed a significant difference in the total population in the PRE vs. DURING phase (3.38 ± 1.09 vs. 3.96 ± 1.56, respectively, *p* < 0.001, Wilcoxon z). The percentage of those who consumed ≥5 meals increased by 23.1% DURING the quarantine compared to the PRE-lockdown phase, and by 2.4% POST-lockdown compared to the PRE-lockdown phase (Figure 5). In addition, there was a significant difference in the number of daily meals between men and women DURING the quarantine (3.73 ± 1.51 vs. 4.11 ± 1.57, respectively, *p* = 0.004, Mann–Whitney U). 

**Figure 6:**
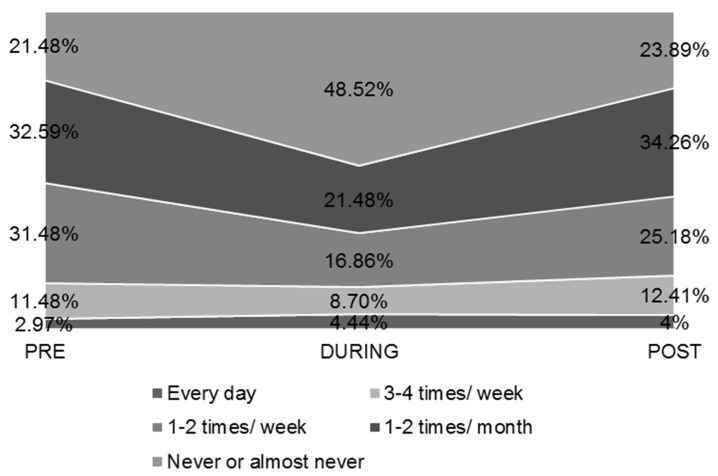
Frequency of alcohol consumption.

The percentage of young men and women who never or almost never consumed alcohol increased by 27.04% DURING the quarantine vs. PRE and by 2.41% POST-lockdown vs. PRE-lockdown (Figure 6). 

There was a significant difference in alcohol consumption between men and women in all the time periods studied (PRE (φ_c_ = 0.710, *p* < 0.001), DURING (φ_c_ = 0.712, *p* < 0.001), POST (φ_c_ = 0.709, *p* < 0.001), Cramer’s V) (Table 6). 

### 3.4. Physical Activity-Exercise

The mean exercise days per week were 3.11 ± 1.61 days in the PRE-lockdown phase. No difference was observed between the phases of the pandemic (Table 7). A significant difference was found in the daily exercise in minutes in the total group in the PRE vs. DURING phase (56.44 ± 50 vs. 46.15 ± 44.29, respectively, *p* < 0.001, Wilcoxon z) in terms of sex. 

Mean exercise days did not differ significantly according to sex (Table 7). However, there was a statistically significant difference between men and women in terms of the kind of physical activity-exercise (φ_c_ = 0.714, *p* < 0.001, Cramer’s V). On the other hand, no significant difference was found between men and women in terms of the type of physical activity-exercise DURING quarantine (φ_c_ = 0.081, *p* = 0.131, Cramer’s V) and the years of physical activity-exercise (*p* = 0.717, Mann–Whitney U), (5.65 ± 4.69 years, total, 5.73 ± 4.72 years in men vs. 5.6 ± 4.68 years in women) (Table 8). 

There was a significant difference in daily exercise time in minutes between men and women in the PRE (65 ± 65.04 vs. 51.25 ± 37.31, respectively, *p* = 0.027, Mann–Whitney U), DURING (54.13 ± 58.39 vs. 41.28 ± 32.02, respectively, *p* = 0.028, Mann–Whitney U), and POST phase (63.1 ± 59.1 vs. 47.77 ± 31.87, respectively, *p* = 0.003, Mann–Whitney U). 

### 3.5. Bivariate Correlations

We assessed for significant correlations between the numerical variables examined in the questionnaire. We observed that the number of cigarettes per day PRE lockdown were significantly correlated with BMI (r = 0.180, *p* = 0.035) and the years of smoking history (r = 0.409, *p* < 0.001). The number of cigarettes per day DURING quarantine was significantly associated with BMI (r = 0.172, *p* = 0.044), years of smoking history (r = 0.451, *p* < 0.001), pack-years (r = 0.750, *p* < 0.001) and the number of cigarettes consumed PRE-lockdown (r = 0.786, *p* < 0.001). The number of cigarettes per day POST-lockdown were significantly correlated with years of smoking history (r = 0.373, *p* < 0.001), pack-years (r = 0.732, *p* < 0.001), the number of cigarettes per day PRE-lockdown (r = 0.847, *p* < 0.001) and the number of cigarettes consumed per day DURING lockdown (r = 0.814, *p* < 0.001). The hours of sleep DURING lockdown were significantly correlated with the hours of sleep PRE-lockdown (r = 0.259, *p* < 0.001) and the number of meals per day PRE-lockdown (r = −0.090, *p* = 0.037). The hours of sleep POST-lockdown were significantly correlated with age (r = −0.136, *p* = 0.002), the hours of sleep PRE-lockdown (r = 0.471, *p* < 0.001), and the hours of sleep DURING lockdown (r = 0.323, *p* < 0.001). The time to sleep arrival PRE-lockdown was significantly correlated with sleep awakenings PRE-lockdown (r = 0.199, *p* < 0.001). The time to sleep arrival DURING quarantine was significantly correlated with the number of cigarettes per day PRE-lockdown (r = 0.600, *p* < 0.001) and the number of sleep awakenings DURING lockdown (r = 0.226, *p* < 0.001). The time to sleep arrival POST-lockdown was significantly correlated with age (r = 0.100, *p* = 0.20), time to sleep arrival PRE-lockdown (r = 0.706, *p* < 0.001), time to sleep arrival DURING lockdown (r = 0.635, *p* < 0.001), sleep awakenings DURING lockdown (r = 0.117, *p* = 0.007), sleep awakenings POST-lockdown (r = 0.201, *p* < 0.001), and the number of meals per day DURING lockdown (r = −0.092, *p* = 0.033). Sleep awakenings PRE-lockdown were significantly correlated with time to sleep arrival PRE-lockdown. Sleep awakenings DURING lockdown were significantly correlated with time to sleep arrival PRE-quarantine (r = 0.128, *p* = 0.003), time to sleep arrival DURING lockdown (r = 0.226, *p* < 0.001), and sleep awakenings PRE-lockdown (r = 0.569, *p* < 0.001). Sleep awakenings POST-lockdown were significantly correlated with hours of sleep DURING lockdown (r = 0.093, *p* = 0.030), time to sleep arrival PRE-lockdown (r = 0.143, *p* = 0.001), time to sleep arrival DURING lockdown (r = 0.107, *p* = 0.13), time to sleep arrival POST-lockdown (r = 0.201, *p* < 0.001), sleep awakenings PRE-lockdown (r = 0.577, *p* < 0.001), and sleep awakenings DURING lockdown (r = 0.563, *p* < 0.001). The number of meals per day PRE-lockdown were significantly correlated with age (r = −0.093, *p* = 0.030) and pack-years (r = −0.193, *p* < 0.001). The number of meals per day DURING lockdown were significantly correlated with BMI (r = 0.097, *p* = 0.024), pack-years (r = −0.135, *p* = 0.002), and the number of meals per day PRE-lockdown (r = 0.486, *p* < 0.001). The number of meals per day POST-lockdown were significantly correlated with pack-years (r = −0.178, *p* < 0.001), number of meals per day PRE-lockdown (r = 0.629, *p* < 0.001), and the number of meals per day DURING lockdown (r = 0.526, *p* < 0.001).

### 3.6. Predictors of POST Lockdown Variables

We performed multiple linear regression analysis to assess for predictors of sleep quality and lifestyle parameters POST the restrictive measures. In the multiple linear regression model, the number of cigarettes before the quarantine (β = 0.339, *p* < 0.001) and the number of cigarettes during the lockdown (β = 0.541, *p* < 0.001) were independently predictive of the number of cigarettes per day POST-lockdown (r^2^ = 0.806). The following variables were predictive of sleep hours POST lockdown: age (β = −0.147, *p* < 0.001), sleep hours before the quarantine (β = 0.376, *p* < 0.001), and sleep hours during the restrictive measures (β = 0.184, *p* < 0.001) (r^2^ = 0.236). For time of sleep arrival POST-lockdown, time of sleep arrival before the lockdown (β = 0.357, *p* < 0.001), time of sleep arrival during the lockdown (β = 0.458, *p* < 0.001), sleep awakenings during the lockdown (β = −0.101, *p* = 0.007), and sleep awakenings after the restrictive measures (β = 0.172, *p* < 0.001) were independently predictive (r^2^ = 0.523). The following variables were predictive for sleep awakenings POST-lockdown (r^2^ = 0.382): time of sleep arrival DURING the lockdown (β = −0.162, *p* < 0.001), time of sleep arrival POST-lockdown (β = 0.230, *p* < 0.001), sleep awakenings PRE-lockdown (β = 0.198, *p* < 0.001), and sleep awakenings POST-lockdown (β = 0.488, *p* < 0.001). For the number of daily meals POST-lockdown, we observed that only the number of daily meals PRE-lockdown (β = 0.468, *p* < 0.001) and the number of daily meals DURING the lockdown (β = 0.311, *p* < 0.001) were independently predictive (r^2^ = 0.462). For exercise days per week POST-lockdown, we demonstrated that time of sleep arrival DURING lockdown (β = −0.157, *p* < 0.001), exercise days per week PRE-lockdown (β = 0.387, *p* < 0.001), and exercise days per week DURING lockdown (β = 0.209, *p* < 0.001) were independently predictive (r^2^ = 0.241).

## 4. Discussion

To our knowledge, this is the first Greek study that investigates sleep and lifestyle changes (smoking, sleep habits, dietary habits, alcohol consumption, physical activity-exercise) that occurred in young adults, aged 18–25 years, due to the COVID-19 pandemic, PRE, DURING, and POST-quarantine. Importantly, we investigated changes in the aforementioned parameters POST-lockdown since most studies focus on behavioral modifications before and during restrictive measures. 

During the quarantine, we observed no significant change in cigarette consumption per day in the total of the subjects, except for the obese subjects who demonstrated an increase. During the lockdown, the percentage of those who smoked 5–10 cigarettes decreased, and the percentage of those who smoked <5 and >10 cigarettes increased. A similar study in Italy in subjects aged 12–86 years showed a decrease in cigarette consumption for the PRE and DURING phases [30], although the age range is different when compared to our study. In our report, we hypothesize that the change in smoking habits may be influenced by social reasons, such as the lack of entertainment and socializing with friends, which changed during the quarantine due to the obligation to stay at home. We examined for changes according to gender and observed that the smoking habits in all three phases of quarantine were different between the two sexes, with men smoking more cigarettes per day in each phase. This explains the fact that while there is no difference in smoking years, there is a statistically significant difference in pack-years between men and women. It is important to note that the percentages of smokers and vaporizers in this study are similar to the general population of smokers and vaporizers in Greece [31], as well as to the general population of smokers worldwide [32].

Regarding smoking during the lockdown, a study in young people at a university in Saudi Arabia showed an increase in smoking [33], while in the general population of Japan there was a 32.1% increase in the number of cigarettes consumed [34]. In Germany, 45.8% of adults increased smoking during the lockdown [35], and in Kuwait the rate of cigarette consumption was much higher among men than women, with more men reporting a significant increase in smoking during the pandemic [36]. A large study of US adults with different racial/ethnic groups observed an increase in cigarette smoking [37]. In another study in Greece the total population studied showed an increase in cigarette consumption, with the exception of young people, who did not [38]. Smoking was reduced in students at a French university [39], in young adults in Sweden [40], and in adults in the United Kingdom [41]. Finally, with regard to smoking, there were no significant changes in the number of cigarettes per day in young adults studying at Bavarian universities [42], in students in the Netherlands [43], and in Polish university students [44], as well as adults in 17 countries in the Middle East and North Africa [45]. There is therefore a heterogeneity in the smoking habit developed by young adults in different countries, which may be due to different prohibitions and restrictive measures imposed by different countries, combined with the differences in culture of young people from different countries, as well as the smoking culture generally in each country. The above attitude of the young smokers seems to be in line with the smoking attitude of adult smokers from different areas, obviously for similar reasons. In this study, we also assessed the smoking behavior in the POST-lockdown phase. The findings show an increase in the number of cigarettes smoked in the total of subjects, but it was more evident in women and in normal weight subjects. 

The restrictive measures and the long stays in the house led to a parallel increase in sleep hours in the entire population of the study in question. DURING the quarantine, the percentage of those who slept <7 h and 7–9 h decreased, while a spectacular increase (23.07%) was recorded in those who slept >9 h. Interestingly, the increase was maintained, to a lesser degree, even after the end of the lockdown. In the POST-quarantine phase there was a significant increase in sleeping hours in the total of the researched population, with the underweight and normal weight subjects showing the greater increase. Moreover, an increase in those sleeping >9 h was also observed in Italy [30] and in the seven UAE Emirates in the general population [46]. Moreover, an increase in sleep hours was observed in a study in Hong Kong for people aged 18–35 years [47], in a study in the general population in Greece, Switzerland, Austria, Germany, France, and Brazil [48], in other studies in Greece [38,49], in adults in America [50] and in the Middle East and North African region [45], while a study showed a decrease in Spain [51] as did a study in five different regions (Austria/Germany, Ukraine, Greece, Cuba, and Brazil) [52]. 

The young people in the researched population, and to an even greater extent, the women, needed more time to fall asleep DURING the quarantine, a need that remained even after the lockdown. A similar increase in sleep time was observed in Italy [53], as well as in an international Google online survey of forty-one research organizations from Europe, North Africa, West Asia, and the Americas [54]. Sleep awakenings increased DURING the quarantine and increased even more in the POST phase. Here, too, women were more affected. 

Restless sleep, morning headaches, morning irritability, and low morning alertness were also parameters that increased significantly DURING the quarantine and remained high even in the POST-quarantine phase. 

DURING the quarantine, young people consumed more meals, with women ahead. An increase in quarantine meals was also observed in the United Kingdom [55], Poland [56], and Brazil [57]. Regarding people who consumed more than five meals per day, their percentage rose by 23.1% DURING the quarantine vs. before the quarantine and by 2.4% POST-lockdown vs. before the lockdown. 

Those who consumed alcoholic beverages every day increased their percentage DURING the quarantine (2.97 vs. 4.44%), while those who never or almost never consumed them doubled their consumption (21.48 vs. 48.52%). An increase in alcohol consumption was recorded in students at a Saudi Arabian university [33], in Germany [35], and in adults in the USA and the UK [37,41]. In contrast, a decrease was observed in college students at a US university [58]. Polish university students did not notice significant changes in alcohol consumption [44]. 

Although exercise days did not differ between the phases of the pandemic, we observed that daily exercise time was reduced DURING the quarantine with women exercising less time than men. Although some studies provide data that support an increase in physical activity during the pandemic [30], most published literature does not verify these results. Early in the pandemic, studies recorded a reduction in the everyday step count and displayed differences in the magnitude of change between countries, possibly due to the large variation in restrictive measures applied worldwide [59]. Studies in Saudi Arabia [33], France [39], the Middle East, the North African region [45], and Brazil [57] also recorded a decrease in physical activity-sport time. Correa et al. [57] assessed exercise levels with the International Physical Activity Questionnaire (IPAQ) and observed reductions in occupational physical activity and elevations in leisure and domestic physical activity. Possible explanations for the reduction in physical activity include the mandatory restriction on movement outside the home due to the lockdown, that prohibited individuals from participating in outdoor sports, attending the gym, or using active transportation to work (i.e., by walking or cycling). Before drawing any definite conclusions, one has to take into account difficulties when examining the level of physical activity, since the current literature is not in agreement about the optimal method used to assess exercise levels [60]. 

Our results have some important clinical and public health implications. We observed an increase in cigarette consumption and a reduction in physical activity that persisted after the expiration of restrictive measures. The aforementioned findings highlight the need for public health system policies that will support healthy lifestyles in young adults in order to restore or to improve the negative impact of the lockdown measures. In the same context, others have advised on the development of psychological intervention programs that aim to limit the adverse effects of COVID-19 pandemic on children’s psychological well-being [26]. 

The present study, besides its strengths, has several limitations. The retrospective design of the present investigation is a limitation since it may be associated with recall bias. We studied subjects aged 18–25 years since they have been underrepresented in previous studies. However, we acknowledge that our results cannot be extrapolated to other age groups. Additionally, the questionnaire was distributed through online social media. This method could not ensure compliance with the parameterization but met the requirements of massive participation of young adults of the Greek population. It was effective in terms of geographical distribution taking into account the restrictive measures due to the pandemic, mainly due to the systematic involvement of young people with the Internet [27,28]. Another limitation of the study includes the fact that changes in several parameters (e.g., BMI, physical activity, sleep duration, time to sleep arrival, etc.) were self-reported and were not subjectively confirmed. In addition, we did not examine for possible changes occurring during the different time phases of the quarantine. Finally, one has to take into account that we assessed changes limited to 3 months following lockdown measures. Future studies are warranted in order to effectively describe whether the changes reported in our research are present after this time frame. 

## 5. Conclusions 

The restrictive measures implied during the COVID-19 pandemic display significant long-term adverse effects on sleep quality as well as on lifestyle parameters, such as smoking habits, alcohol consumption, and physical activity. With the exception of the smoking habits and the consumption of alcoholic beverages that differ greatly between different countries and age groups, we observe that the other parameters were affected in a similar way around the world during and/or because of quarantine. We observed that young people in general increased their meal consumption, sleeping hours, their sleep onset time, while their daily exercise time decreased significantly. Our results support the need for the development of public health policies that will support healthy lifestyles in this population during the recovery from the COVID-19 pandemic. 

It is worth noting that in the present study, some additional parameters related to the quality of sleep were exclusively investigated, with significant results. It was observed that sleep awakenings, restless sleep, as well as morning irritability and headache showed a significant increase during the quarantine, parameters that were maintained even after the end of the lockdown measures in the study population. Further studies should be designed to elucidate whether these changes persist over longer time spans. 

## Figures and Tables

**Table 1:** Demographic characteristics of the study population.

Variable	Total N = 540	Males N = 201 (37.2%)	Females N = 339 (62.8%)	*p*-Value ^a^
Age, (years)	21.2 ± 2.3	21.44 ± 2.32	21.09 ± 2.29	0.089
Weight, (kgr)	69.9 ± 15.8	79.45 ± 15.11	64.37 ± 3.43	<0.001
Height, (cm)	171 ± 9.3	179.61 ± 7.17	165.98 ± 6.33	<0.001
BMI, (kg/m^2^)	23.8 ± 4.5	24.58 ± 4.15	23.35 ± 4.66	<0.001
Underweight	38 (7%)	7 (18.4%)	31 (81.6%)	
Normal	336 (62.2%)	122 (36.3%)	214 (63.7%)	
Overweight	114 (21.1%)	51 (44.7%)	63 (55.3%)	
Obese	52 (9.6%)	21 (40.4%)	31 (59.6%)	

^a^*p*-value corresponds to comparison of males vs. females with the use of the Mann–Whitney test.

**Table 2:** Smoking history of smokers.

	Total N = 540	Males N = 201 (37.2%)	Females N = 339 (62.8%)	*p*-Value
Variable				
Smoker	138 (25.5%)	66 (47.8%)	72 (52.2%)	
Years of smoking	5.07 ± 2.49	5.32 ± 2.71	4.84 ± 2.26 *	0.296 ^a^
Pack-Years	0.73 ± 1.76	1.18 ± 2.33	0.46 ± 1.24	0.001 ^a^
Vaping	12 (2.2%)	8 (66.7%)	4 (33.3%)	
Years of vaping	2.63 ± 1.4	2.2 ± 1.3	3.33 ± 1.52	0.3 ^b^
Smoker + Vaping	8 (1.5%)	1 (12.5%)	7 (87.5%)	
Years of smoking	2.53 ± 1.5	3 ± 1.41	2.38 ± 1.55	0.492 ^b^
Years of vaping	2.11 ± 1.05	2 ± 1	2.25 ± 1.25	0.749 ^b^
Former smokeror /and vaping	12 (2.2%)	5 (41.7%)	7 (58.3%)	
NOΝ SMOKERS	370 (68.4%)	121 (32.7)	249 (67.3%)	

* *p* = 0.001 (comparison of males vs. females), ^a^ Mann–Whitney U test, ^b^ *t*-test.

**Table 3:** Cigarettes per day.

	Total	Males	Females	*p*-Value ^a^
PRE	10.10 ± 5.84	12.18 ± 6.11	8.19 ± 4.88	<0.001
DURING	10.76 ± 8.5	12.67 ± 8.96	9.01 ± 7.7	0.009
POST	11.25 ± 6.72	12.89 ± 6.8	9.74 ± 6.32	0.005

^a^*p*-value corresponds to the comparison of males vs. females with the use of the Mann–Whitney test.

**Table 4:** Sleep–wake habits of the study population.

	Total N = 540	Males N = 201 (37.2%)	Females N = 339 (62.8%)
Restless sleep			
PRE	154 (28.51%)	52 (25.9% ^a^)	102 (30.1% ^b^)
DURING	235 (43.51%)	67 (33.3% ^a^)	168 (49.6% ^b^)
POST	266 (49.26%)	80 (39.8% ^a^)	186 (54.9% ^b^)
Difficulty in getting up			
PRE	278 (51.4%)	91 (45.3% ^a^)	187 (55.2% ^b^)
DURING	292 (54%)	90 (44.8% ^a^)	202 (59.6% ^b^)
POST	295 (54.5%)	97 (48.3% ^a^)	198 (58.4% ^b^)
Morning headache			
PRE	54 (10%)	13 (6.5% ^a^)	41 (12.1% ^b^)
DURING	139 (25.75%)	31 (15.4% ^a^)	108 (31.9% ^b^)
POST	133 (24.6%)	32 (15.9% ^a^)	101 (29.8% ^b^)
Morning irritability			
PRE	127 (23.52%)	37 (18.4% ^a^)	90 (26.5% ^b^)
DURING	228 (42.22%)	71 (35.3% ^a^)	157 (46.3% ^b^)
POST	184 (34.07%)	63 (31.3% ^a^)	121 (35.7% ^b^)

^a^ males, ^b^ females.

**Table 5:** Morning alertness of the study population.

	Total N = 540	Males N = 201 (37.2%)	Females N = 339 (62.8%)
High			
PRE	91 (16.85%)	33 (16.4%)	58 (17.1%)
DURING	64 (11.85%)	22 (10.9%)	42 (12.4%)
POST	97 (17.96%)	31 (15.4%)	66 (19.5%)
Medium			
PRE	347 (64.29%)	133 (66.2%)	214 (63.1%)
DURING	221 (40.93%)	97 (48.3%)	124 (36.6%)
POST	317 (58.70%)	124 (61.7%)	193 (56.9%)
Low			
PRE	102 (18.86%)	35 (17.4%)	67 (19.8%)
DURING	255 (47.22%)	82 (40.8%)	173 (51%)
POST	126 (23.34%)	46 (22.9%)	80 (23.6%)

**Table 6:** Alcohol consumption of the study population.

	Total N = 540	Males N = 201 (37.2%)	Females N = 339 (62.8%)
Every day			
PRE	16 (2.97%)	6 (3%)	10 (2.9%)
DURING	24 (4.44%)	8 (10.9%)	16 (4.7%)
POST	23 (4.26%)	12 (6%)	11 (3.2%)
3–4 times/week			
PRE	62 (11.48%)	28 (13.9%)	34 (10%)
DURING	47 (8.70%)	25 (12.4%)	22 (6.5%)
POST	67 (12.41%)	27 (13.4%)	40 (11.8%)
1–2 times/week			
PRE	170 (31.48%)	63 (31.3%)	107 (31.6%)
DURING	91 (16.86%)	35 (17.4%)	56 (16.5%)
POST	136 (25.18%)	46 (22.9%)	90 (26.5%)
1–2 times/month			
PRE	176 (32.59%)	57 (28.4%)	119 (35.1%)
DURING	116 (21.48%)	45 (22.4%)	71 (20.9%)
POST	185 (34.26%)	67 (33.3%)	118 (34.8%)
Never or almost never			
PRE	116 (21.48%)	47 (23.4%)	69 (20.4%)
DURING	262 (48.52%)	88 (43.8%)	174 (51.3%)
POST	129 (23.89%)	49 (24.4%)	80 (23.6%)

**Table 7:** Activity-exercise (days per week) of the study population. The comparison was performed with the use of Mann–Whitney U test.

	Total N = 433	Males N = 164 (37.87%)	Females N = 269 (62.13%)	*p*-Value
PRE	3.11 ± 1.61	3.25 ± 1.66	2.98 ± 1.56	0.37
DURING	3.18 ± 2.11	3.47 ± 2.32	2.92 ± 1.88	0.22
POST	3.08 ± 1.61	3.33 ± 1.61	2.85 ± 1.60	0.06

**Table 8:** Physical activity-exercise of the study population.

	Total N = 540	Males N = 201 (37.2%)	Females N = 339 (62.8%)
**Kind**			
Fitness center	100 (18.51%)	49 (24.4%)	51 (15%)
Biking/cycling	30 (5.56%)	13 (6.5%)	17 (5%)
Walking	188 (34.81%)	57 (28.4%)	131 (38.6%)
Other	115 (21.3%)	45 (22.4%)	70 (20.6%)
No activity/exercise	107 (19.82%)	37 (18.4%)	70 (20.6%)
**Type**			
Free	305 (56.48%)	108 (53.7%)	197 (58.1%)
Instructed	128 (23.7%)	56 (27.9%)	72 (21.2%)
No activity/exercise	107 (19.82%)	37 (18.4%)	70 (20.6%)

## Data Availability

The data presented in this study are available on request from the corresponding author. The data are not publicly available due to privacy.

## Supplementary Materials

The following supporting information can be downloaded at: https://www.mdpi.com/article/10.3390/ijerph191912333/s1. Table S1. Questionnaire of the study.
