# Supplementary material for: Long-Term Impact of COVID-19 Pandemic in Sleep Quality and Lifestyle in Young Adults
Source: Int J Environ Res Public Health. 2022 Sep 28;19(19):12333. doi: 10.3390/ijerph191912333 (PMC9564795; doi:10.3390/ijerph191912333)
Supplement: Supplementary file 1 [file ijerph-19-12333-s001.zip › ijerph-1939954-supplementary.pdf]

International Journal of
Environmental Research and Public Health

MDPI

Table S1. Questionnaire of the study.

Demographics

**Age (years): …….**	**Gender:**<br>**Male** □<br>**Female** □	**Weight in Kilograms:……………….**<br>**.**<br>**Height in cm:**<br>**………………..**	**Smokinghistrory:**<br>**Smoker:** □<br>**Vaper:** □<br>**Smoker and vaper:** □<br>**Ex smoker and/or vaper:** □<br>**No smoker and no vaper:** □

**If smoker or smoker and vaper:**<br>Years of smoking: ………….<br>Cigarettes per day 3 months prior to lockdown: ………….<br>Cigarettes per day during lockdown: ………….<br>Cigarettes per day 3 months following lockdown:………….	**If vaper or smoker and vaper…**<br>Years of vaping: ………….<br>ml per day 3 months prior to lockdown: ………….<br>ml per day during lockdown: ………….<br>ml per day 3 months following lockdown:………….

**Sleeping habits**	How many hours did you sleep per day**3 months prior to lockdown: ……………………………..**<br>How many hours did you sleep per day**during lockdown: ……………….**<br>How many hours did you sleep per day**3 months following lockdown:……………………………..**<br>How many times in minutesdid you need until you fell asleep at night (per day)**3 months prior to lockdown: ………………………..**<br>How many time in minutes did you need until you fell asleep at night (per day)**during lockdown: …………………………….**<br>How many time in minutes did you need until you fell asleep at night (per day)**3 months following lockdown::…..**<br><br>How many times did you wake per night **day 3 months prior to lockdown: ……………………………..**<br>How many times did you wake per night **dayduring lockdown: ……………….**<br><br>How many times did you wake per night **day3 months following lockdown: ………………………..**<br><br>Did you experience restless sleep **3 months prior to lockdown:**Yes No<br>Did you experience restless sleep**during lockdown:**Yes No

	Did you experience restless sleep**day3 months following lockdown:**Yes No<br><br>Did you experience difficulty in waking up **3 months prior to lockdown:** Yes No<br>Did you experience difficulty in waking up **during lockdown:** Yes No<br><br>Did you experience difficulty in waking up **3 months following lockdown:** Yes No<br><br>Did you experience morning headaches**3 months prior to lockdown:** Yes No<br>Did you experience morning headaches**during lockdown:** Yes No<br><br>Did you experience morning headaches**3 months following lockdown:** Yes No<br><br>Did you experience irritability during the morning **3 months prior to lockdown:** Yes No<br>Did you experience irritability during the morning **during lockdown:** Yes No<br><br>Did you experience irritability during the morning **3 months following lockdown:** Yes No

**Dietary habits**	Number of daily meals**3 months prior to lockdown: ……………………………..**<br>Number of daily meals**during lockdown: ……………….**<br>Number of daily meals**3 months following lockdown: ………………………..**<br><br>How often did you consume alcohol **3 months prior to lockdown:**<br>Every day: □<br>3–4 times/week: □<br>1–2 times/week: □<br>Never or almost never: □<br><br>How often did you consume alcohol **during lockdown:**<br>Every day: □<br>3–4 times/week: □<br>1–2 times/week: □

	Never or almost never: □<br><br>How often did you consume alcohol **3 months following lockdown:**<br>Every day: □<br>3–4 times/week: □<br>1–2 times/week: □<br>Never or almost never: □
**Physical activity-exercise**	**Type of physical activity-exercise**<br>Gym: □<br>Cycling: □<br>Walking: □<br>Other: □<br>No activity: □
	Kind of physical activity-exercise<br>Free: □<br>Instructed: □
	**Years of physical activity-exercise: ……………………**
	How many days per week did you perform physical activity-exercise **3 months prior to lockdown: ……………………………..**<br><br>How many days per week did you perform physical activity-exercise **during lockdown: ……………….**<br>How many days per week did you perform physical activity-exercise **3 months following lockdown: ………………………..**
	Time in minutes of physical activity-exercise per day3 months prior to lockdown: ……………………………..<br>Time in minutes of physical activity-exercise per day**during lockdown: ……………….**<br><br>Time in minutes of physical activity-exercise per day **3 months following lockdown**: ……………….
